# Methods for Extracellular Vesicle Isolation: Relevance for Encapsulated miRNAs in Disease Diagnosis and Treatment

**DOI:** 10.3390/genes16030330

**Published:** 2025-03-12

**Authors:** Maria Ljungström, Elisa Oltra

**Affiliations:** 1 Escuela de Doctorado, School of Health Sciences, Catholic University of Valencia, 46001 Valencia, Spain; maria.ljungstrom@mail.ucv.es; 2 Department of Pathology, School of Health Sciences, Catholic University of Valencia, 46001 Valencia, Spain

**Keywords:** extracellular vesicle, exosome, microRNA, isolation, diagnosis, treatment

## Abstract

Extracellular vesicles (EVs) are nanovesicles that facilitate intercellular communication by carrying essential biomolecules under physiological and pathological conditions including microRNAs (miRNAs). They are found in various body fluids, such as blood, urine, and saliva, and their levels fluctuate with disease progression, making them valuable diagnostic tools. However, isolating EVs is challenging due to their small size and biological complexity. Here, we summarize the principles behind the most common EV isolation methods including ultracentrifugation, precipitation, immunoaffinity, sorting, ultrafiltration, size exclusion chromatography, and microfluidics while highlighting protocol strengths and weaknesses. We also review the main strategies to identify and quantify circulating miRNAs with a particular focus on EV-encapsulated miRNAs. Since these miRNAs hold special clinical interest derived from their superior stability and therapeutic potential, the information provided here should provide valuable guidance for future research initiatives in the promising field of disease diagnostic and treatment based on EV-encapsulated miRNAs.

## 1. Introduction

Extracellular vesicles (EVs) and their encapsulated microRNAs (miRNAs) are emerging as crucial tools for the diagnosis and treatment of human disease [1,2,3,4,5,6,7,8,9,10,11].

EVs are nanovesicles that originate from cells with a role in intercellular communication [12,13,14]. They carry a variety of biomolecules which play significant roles in both physiological and pathological processes [15]. Research has shown that EVs are involved in the disease progression, for example, being associated with tumor growth stage [16,17,18] or to degeneration in neurodegenerative disorders [2,3]. Their ability to carry information from parental cells catalogues them as mediators to influence the behavior of target cells, becoming critical in the understanding of human physiology as well as in the mechanisms of disease [13,14,19]. These findings unleashed a crusade to intensively explore EVs’ potential as biomarkers for early disease detection and for disease monitoring. EVs can also provide insights into drug efficacy and individuals response to treatment, highlighting their wide potential as tools in clinical settings [1,3,8]. Their biomarker capacity extends to guiding therapy in cardiovascular and other diseases challenged with post-treatment recidivism, such as cancers, neurodegenerative conditions or tissue repair treatments [2,4,5,10,20,21,22,23].

EVs can be found in many body fluids, including blood, urine, saliva, and others, which provide the media for EV-based long-range cell–cell communication [6,8], thus becoming valuable for liquid biopsy minimally invasive approaches in the development of precision medicine programs, and in the accurate prediction of patient prognosis and treatment outcomes [9].

On another side, microRNAs or miRNAs are small non-coding RNA molecules, typically 19–25 nucleotides in length (most mature miRNAs have 22 nucleotides) [24], which regulate gene expression at the post-transcriptional level [25]. They play important roles in both physiological and pathological processes, and their dysregulation has also been observed in numerous diseases, making them attractive targets for diagnosis and treatment methods as well [3,26]. The encapsulation of miRNAs within EVs provides an additional layer of stability and specificity for the action of miRNAs [8,9,10]. EV double-membrane protects miRNAs from enzymatic degradation, preserving their integrity in circulating fluids, with EV surface markers and cargoes often reflecting the health status of their parental cells, thus providing insights into tissue or cell-type origin of the pathology and its associated mechanisms [26,27,28,29,30].

While the potential of EVs and their miRNA contents seems promising in future clinical applications, there are still challenges to standardize their research and their use in the clinic. According to the latest guidelines provided by MISEV 2023, there is no golden-standard method to isolate or detect EVs [31]. Depending on the origin of the sample, the EVs’ subtype or the downstream purpose, the most appropriate technique or approach to isolate and detect the target EVs is different for each experimental circumstance [31].

This review provides an updated overview of the main methods used for the isolation of EVs from biofluids and for the downstream inspection of them and the miRNAs contained within, while highlighting associated advantages and disadvantages for each method. It also provides examples of EV-encapsulated miRNAs catalogued as disease biomarkers while summarizing the methods used for their identification. This information may serve as valuable guidance for future research initiatives in the promising field of disease diagnosis and treatment based on EV-encapsulated miRNAs.

## 2. EVs: Biogenesis, Function, and Clinical Potential

The first observation of EVs seems to trace back to the 1940s when small particles shedding off platelets were noticed while studying blood cells under a microscope; particles that were later coined “platelet dust” by Wolf (1967) [32,33]. At this time, there was no clue of the universality and relevance of this phenomenon. In 1969, EVs were identified while describing the process of bone formation. Those EVs were named matrix vesicles, seemingly helping in the process of the hardening of bones, which led researchers to propose their involvement in an important physiological process [34,35].

Then, EVs were found to be released also by intestinal cells, suggesting that EVs may come from different cell types, possibly representing a universal process. In the 1980s, EVs were detected in cellular cultures and informed as particles with a virus-like morphology, indicating that EVs may display various appearances [36,37]. During this decade, vesicles were discovered also in semen, which received the name prostasomes. These and other findings pointed to EVs being present in many or perhaps all body fluids possibly performing different functions [38], with implications in pathological processes [39].

In 1983, it was found that some of the vesicles, nowadays named exosomes, come from inward budding processes, driving the formation of larger structures within cells or multivesicular bodies (MVBs) [40]. In the late 1990s, a significant breakthrough occurred when researchers discovered that exosomes could present antigens, triggering immune responses, representing a major step in understanding how EVs can influence the immune system [41].

In 2006 and 2007, EVs were found to contain RNA, including microRNAs [42]. This discovery sparked great interest because it showed that EVs could carry genetic information acting as directional communication vehicles between cells in a new method of long-range cell-to-cell communication. Since then, EVs have been isolated from many different types of cells and body fluids, such as saliva, urine, plasma, CSF, and breast milk, among others, leading to the current general acceptance that EVs spread throughout our bodies and are involved in many health-relevant functions [6,8,9].

### 2.1. Biogenesis of EVs

**Figure 1:**
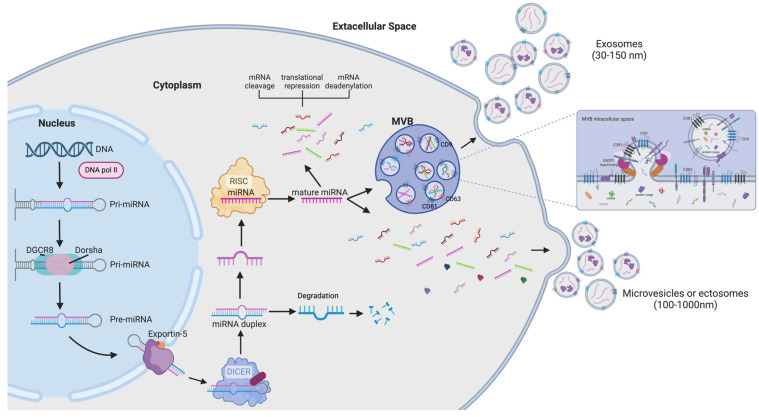
EV/miRNA biogenesis: miRNA genes are transcribed as pri–miRNAs by RNA polymerase II (Pol II) [53], then cleaved by DROSHA and DGCR8 to obtain the pre–miRNAs. Processed pre–miRNAs are then exported to the cytoplasm by exportin 5 and further processed by DICER1 [53,54]. One strand of the mature miRNA is then loaded into a miRNA–induced silencing complex (miRISC) containing DICER1 and Argonaute proteins. Mature miRNAs may mediate gene suppression by targeting mRNA degradation or/and translational repression; or can be encapsulated in EVs [53,54]. MVBs merge with the plasma membrane to release exosomes regulated by lipid–dependent pathways and the ESCRT machinery (colored in pink and orange). Detail of exosome formation by the endosomal network derived in an MVB is shown. Also, the right part of the figure illustrates microvesicles merging by plasma membrane fission, as previously described [12,49,51] (created with Biorender).

EVs are heterogeneous membrane-bound particles released by practically all cell types. They are classified into three main groups based on their size and origin: exosomes (30–150 nm), microvesicles or ectosomes (100–1000 nm), and apoptotic bodies (500–2000 nm) [43,44]. Exosome biogenesis begins within endosomes, which develop into multivesicular bodies (MVBs) (Figure 1) [13,43,44,45,46,47], which can either fuse with lysosomes for degradation or merge with the plasma membrane to release exosomes [48]. Key regulators of this process include the endosomal sorting complexes required for transport (ESCRT) machinery, tetraspanins, including CD9, CD63, and CD81, and lipid-dependent pathways such as ceramide-mediated mechanisms [49,50,51]. Microvesicles, by contrast, originate by outward budding of the plasma membrane, driven by cytoskeletal reorganization and lipid redistribution processes (Figure 1) [12,43,44,49,52].

### 2.2. EV Function

EVs mediate long-range intercellular communication by transferring their cargo to target cells (directional transfer), which include proteins, lipids, DNA, and RNA (miRNAs included), exerting their influence in numerous biological processes such as immune modulation [55], angiogenesis [16,18], cellular proliferation [56], and many others. EVs also play roles in disease by promoting tumor progression through metastasis, mediating immune evasion, drug resistance, and others. For example, cancer-derived EVs can modify the tumor microenvironment by delivering oncogenic factors to stromal cells [8,15,16,17,22].

### 2.3. EVs Clinical Potential

EVs are being studied as therapeutic delivery vehicles due to their natural biocompatibility, ability to evade immune surveillance, and efficient cellular uptake. Engineered EVs can deliver therapeutic molecules, including miRNAs, siRNAs, proteins, and drugs, to target cells [5,11,57]. For instance, mesenchymal stem cell (MSC)-derived EVs have shown promising results in treating inflammatory and neurodegenerative diseases by delivering anti-inflammatory cytokines or neuroprotective factors [58,59]. EV-based therapies for cancer are also being explored, with preclinical studies showing success in delivering chemotherapeutics and suppressing tumor growth [60]. As mentioned earlier, EVs are being considered as non-invasive biomarkers for diagnostic purposes due to their wide presence in biological fluids such as blood, urine, saliva, or cerebrospinal fluid (CSF), which can be obtained less invasively than solid tissues [6,13].

Sample collection, pre-processing, and storage of EVs are important considerations to obtain quality samples and data [61]. The impact of storage strategy on EVs has been extensively studied, and recommendations seem to depend on sample origin and downstream analyses or use. MISEV 2023 guidelines provide specific recommendations according to biofluid type [31]. Freezing cycles damage EVs; therefore, avoiding repeated freezing/thawing cycles helps reduce EV loss and experimental artifacts [44,61,62].

The standardization of simplified EV isolation techniques, allowing the translation of EV potential for either diagnosis or treatment, is being intensively pursued. The current main protocols used for the isolation of EVs include ultracentrifugation (UC), precipitation, immunoaffinity capture, EV sorting, ultrafiltration (UF), size exclusion chromatography (SEC), and microfluidics [31,63]. Downstream analyses of isolated EVs, including proteomic and RNA sequencing, enable the identification of disease-specific signatures. However, in this review, we will concentrate on the detection of miRNAs which in addition to their diagnostic value are envisioned as potential therapeutic options.

## 3. Methods to Isolate EVs

EVs can vary in size, cargo, and surface markers, which can help in their cataloguing and influence their optimized isolation methods.

Isolating EVs is a complex task due to their small size and the variety of types present in the fluid of interest. Nowadays, there is no unique standardized optimal method for EV isolation since each method presents strengths and weaknesses. Different isolation methods favor the purification of certain types of EVs over others, and thus, the optimized method may need tailoring according to disease type. In addition, contaminants like protein clumps or other particles that are not EVs may complicate their downstream analysis [64,65,66,67]. Understanding the particularities of each method seems crucial for the translation of methods into a clinical setting. The main EV isolation methods used in research are described in this section.

### 3.1. UC

UC is the most widely used exosome separation technique. The conventional UC approach relies on the sedimentation principle [68,69]. And in research, it is commonly used to isolate EVs from nearly all biofluid samples including plasma, breast milk, CSF, amniotic fluid, urine, aqueous humor, and cell culture lines [12,69].

UC allows upscaling of the process [70]. However, the EV structure may be disrupted by applying large shear forces during UC. The main drawbacks are EV loss, fusion, distortion, and co-isolation of contaminants like proteins or other complexes [71,72]. While effective, it often requires larger volumes compared to other techniques due to the low abundance of EVs in biofluids and UC equipment capacity [73]. Differential UC (dUC) and density gradient UC (DGUC) are two subtypes of the technique.

#### 3.1.1. dUC

**Figure 2:**
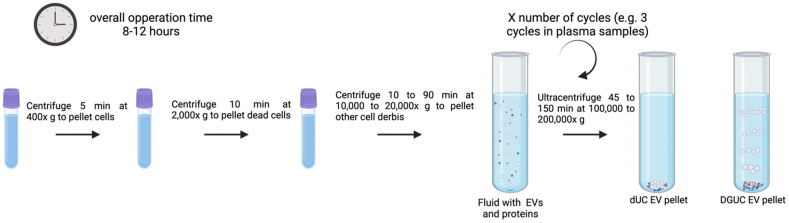
EV isolation using UC, as previously described. It requires several ultracentrifugation cycles to pellet or isolate EVs. Specific UC speed and time depends on sample density, temperature, and rotor used. Time, number of cycles, and speed will vary depending on the type of UC performed, dUC or DGUC. Generally, the process for all the fluids containing EVs consists of an initial centrifugation step to remove cells and large contaminants contained in the fluid followed by two or three additional centrifugations to remove other debris and pellet the EVs [69,70,71,72,73,74,75] (created with Biorender).

dUC is commonly used to purify exosomes from other vesicles, proteins, and cell debris. Often also called UC, it uses serial rounds of centrifugation with a number of cycles and speed depending on the vesicle size range under isolation [74,75]. dUC requires an intense hands-on approach for the process of programming spin cycles and running them to separate pellets and supernatants. It also demands larger sample quantities since significant losses are expected by repeated cycles of sample transfer between tubes [76]. The principle of this method is the use of a low centrifugal force to remove big particles and cell debris to then isolate purified EVs from supernatants at higher forces. To pellet cells, big detritus, apoptotic bodies, and clumps of biopolymers, biological fluid samples are first centrifuged at 10,000–20,000× *g* for 10 to 90 min. The exosomes are extracted from the supernatant by one to three cycles of centrifugation of 45 to 150 min/each at 100,000–200,000× *g*, as illustrated in Figure 2 [31]. All centrifugation steps must be performed at 4 °C. The exosome pellets are then resuspended in sterile filtered phosphate-buffered saline (PBS) buffer or another stable buffer and stored for further analysis at −80 °C for extended periods or at 4 °C for their short-term use.

This method’s primary benefits are its low processing costs, capacity to handle large sample volumes (1–25 mL), simultaneous separation of multiple EV samples, and absence of additional chemicals needed for the procedure.

This process is time-consuming and is dependent on rotor type and its characteristics, as well as the temperature and viscosity of the starting liquid (properties of the biofluid under study) [77]. Limitations include heterogeneity of fluid composition and the presence of many vesicles of comparable sizes and protein aggregates that can co-form at 100,000× *g*. Additionally, exosome aggregation occurs at high centrifugal speeds over extended periods.

Figure 2 illustrates a typical UC process as described previously [69,70,71,72,73,74,75] for different biofluids (plasma, urine, cell culture media, etc.) with volumes ranging from 1 to 25 mL and estimated overall operation times of 8 to 12 h.

#### 3.1.2. DGUC

DGUC, sometimes referred to as isopycnic UC, uses a number of solvents of different densities displayed discontinuously to trap exosomes between different layers [26,31]. As illustrated in Figure 2, DGUC centrifugation enhances particle separation efficiency according to their buoyant density values, enabling separation of subcellular components [73]. DGUC centrifugation separates exosomes based on the variations in their mass density and size relative to other constituents in shorter time and reduced number of cycles as compared to dUC.

It is considered a practical method for EV subtype isolation. However, its success depends on the type of rotor and its properties, as well as the initial liquid’s temperature and viscosity. Additional cleaning procedures are required since the EVs will be isolated in a solution that is typically incompatible with downstream analysis. Standard centrifugation methods must be optimized based on the characteristics of the origin of biofluid samples and the rotor used [77].

### 3.2. Precipitation

Precipitation-based techniques mostly use highly hydrophilic polymers, such as polyethylene glycol (PEG) or its derivatives, to chemically precipitate EVs [78]. The process of EV precipitation relies on the presence of PEG, which indices crowding and exclusion effects, reducing the solubility by bringing exosomes closer to each other while disrupting the hydration layers surrounding exosomes [79]. In this approach, samples are co-incubated with 8–12% 6 kDa PEG solution at 4 °C overnight. Experimental evidence shows that adding positively charged protamine molecules may promote vesicle aggregation during the incubation phase [80]. To separate EVs from serum, Helwa et al. compared UC and three commercial PEG precipitation kits: the miRCURY Exosome Kit (Qiagen, Hilden, Germany), the Total Exosome Isolation Reagent (TEIR; Invitrogen, Waltham, MA, USA), and ExoQuick-Plus (System Biosciences, Palo Alto, CA, USA) [68]. The quantity of EVs recovered by differential centrifugation was approximately 130 times lower than the quantity isolated with commercial kits. Except for TEIR, which produced yields notoriously higher than miRCURY, the yields of all other commercial kits were reported to be similar [68]. The ExoQuick manufacturer protocol states that the samples used in tests are precleared to remove cells and cellular debris. The cleared solution is then incubated with the appropriate volume of ExoQuick for 0.5–12 h, depending on the sample origin. After incubation, the solution is centrifuged for 20 min at 16,000× *g* at 4 °C to pellet the EVs. The pellet can be resuspended in the appropriate sample buffer, such as sterile-filtered PBS, and analyzed or stored at −80 °C [81]. The final preparation barely contains any precipitating agent, but since the pellet is collected by high-speed centrifugation, it frequently contains clumps of proteins and other contaminants.

The precipitation-based isolation technique does not require specialized equipment, is quick, simple, and affordable, and only requires a modest sample size. Since it does not damage EVs and does not call for any extra equipment, precipitation is recognized as the most straightforward and quickest technique for isolating EVs. For clinical research, these features seem the most desirable.

**Figure 3:**
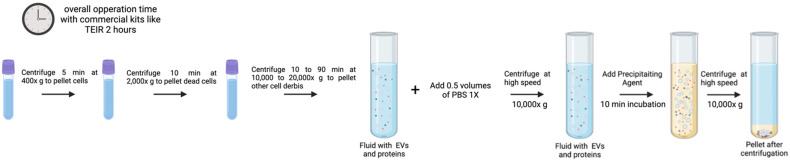
General precipitation–based protocol for EV isolation following the manufacturer’s “Total Exosome Isolation (from plasma) Invitrogen” methodology. Although the previously stated PEG precipitation process has the same bases, the time setting would change due to the overnight precipitation step. Thus, TEIR takes two hours to operate, while a traditional PEG–precipitation technique might take up to twenty–four hours [68,78,79,80] (created with Biorender).

The poorer product purity is a drawback of the method [82,83]. High protein contamination limits the use of precipitation techniques to analyze EV samples under certain conditions. Furthermore, exosomes that have been separated by precipitation techniques could include biopolymers that may interfere with downstream assays. An efficient pre-filtration step with a 0.22 μm filter or a post-precipitation purification procedure that involves additional centrifugation, or an additional 0.22 μm filtration could reduce contamination with non-EV pollutants [84]. Exosome isolation from human biological fluids has greatly improved thanks to upgrades and improvements to the original polymer precipitation method. Two commercial kits that use polymer precipitation with an increased yield and purity in comparison to those formerly mentioned are ExoQuick-Plus (System Biosciences, Palo Alto, CA, USA) and ExoEasy (Qiagen, Hilden, Germany) [85]. These commercial kits provide a practical alternative together with the Total Exosome Isolation kit (Invitrogen, Waltham, MA, USA) (Figure 3) for quick and labor-saving methods to isolate EVs, but their lack of specificity imposes limitations for downstream applications.

Figure 3 illustrates a graphical summary of a commercial EV isolation kit as described by the “Total Exosome Isolation (from plasma) Invitrogen” manufacturer protocol. This protocol has been optimized for plasma samples, while the described PEG precipitation protocol can be used in all types of fluids. For this process, the overall operation time is 2 h, and for a conventional PEG-precipitation protocol, the time increases to up to 24 h [68,78,79,80]. The volume of work can be easily scaled, but the optimal working sample volume goes from 0.1 to 1 mL [68].

### 3.3. Immunoaffinity

**Figure 4:**
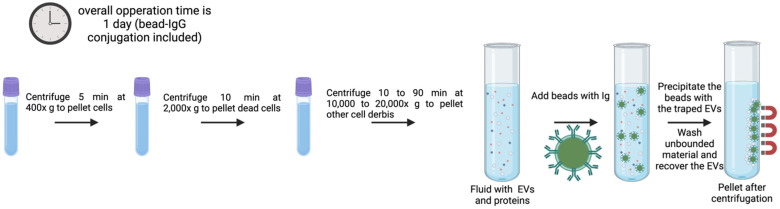
Generalized example of immunoaffinity protocol for EV isolation with magnetic beads as solid phase. The protocol timing may change depending on the target concentration; however, the overall estimated time of incubation is from 1 h to overnight. EVs from all types of biofluids can be isolated using immunoaffinity. There is no strict working volume value, but it needs to be optimized according to sample viscosity [67] (created with Biorender).

These strategies are based on the identification of specific EV subsets according to their surface protein composition. Certain proteins and receptors are commonly present in some EV populations [86], offering a chance to anchor antibodies for immunoaffinity-based EV isolation (Figure 4) [87]. Immunoaffinity-based EV capture may in theory be used towards any protein or cell membrane component that is expressed on EV membranes and that does not count with soluble equivalents in the fluid. Transmembrane proteins, heat shock proteins, platelet-derived growth factor receptors, fusion proteins (such as flotillins, annexins, and GTPases), lipid-related proteins, and phospholipases are some of the exosome markers that have been identified over the last few decades [87,88,89,90]. Databases of proteins contained in vesicles, such as Exocarta, Vesiclepedia, EVpedia, ExoRBase or EV-TRACK, were built for the study of EVs and may facilitate the selection of particular subpopulations by immunoaffinity. EVs have a wide variety of markers on their membranes, and some of the most generally used for immunoaffinity of exosomes are the tetraspanins CD9, CD63 or CD81, although not all EVs contain these tetraspanins concomitantly on their surface. Therefore, with this technique, it is not possible to isolate all EV types present in a sample; it rather focuses on the isolation of specific subpopulations. Immunoaffinity-based isolation techniques preserve EV functionality, allowing the study of the effects (potential functions) of EV subtypes [91].

In addition to the mentioned tetraspanins, other transmembrane proteins like Rab5, CD82, annexin, and Alix have been described for selective exosome isolation [92,93]. As a result, several exosome isolation products have been developed, such as the Exosome Isolation Kit CD81/CD63 (Miltenyi Biotec, Bergisch Gladbach, Germany), the Exosome Isolation and Analysis Kit (Abcam, Cambridge, UK), and the Exosome-human CD63 isolation reagent (Thermo Fisher Scientific, Waltham, MA, USA). Immunoaffinity capture offers the opportunity to classify distinct EV subpopulations of certain origin through their associated surface markers.

Solid matrices to attach antibodies for immunoaffinity include agarose and magnetic beads, plastic plates, and different kinds of microfluidic devices, with the latter being under intensive development in recent years [67]. The most often used are submicron-sized magnetic particles (commonly known as magnetic beads) (Figure 4), the reasons being their broad binding surface, constituting near-homogeneous processes with excellent capture efficiency and sensitivity. They offer the possibility of handling very large initial sample volumes, enabling upscaling or downscaling for specific applications [67]. Furthermore, its facility of operation and automatization facilitates its translation into potential diagnostic platforms when using disease-specific antibodies and magnetically induced cell sorting [94]. A prior study showed that exosomes released by tumors could be specifically isolated, both from culture medium of tumor-derived cell lines and clinical samples, by using magnetic beads coated with antibodies recognizing EpCAM (a surface marker overexpressed on tumor-derived exosomes) [95,96]. Gathering exosomes of a particular origin offers crucial information about their parental cells which may be valuable for diagnosis and treatment options [95,96,97].

Although immunoaffinity-based EV isolation methods guarantee high-purity exosome isolation by a simple process, the collected EV biological functions may be permanently impacted by the non-neutral pH and non-physiological elution buffers used in the process (to elute vesicles from the antibodies). Denatured EV samples are not suitable for exosome-based functional research and other therapeutic applications, although they are typically acceptable for some diagnostic reasons (e.g., by evaluating the genetic and protein contents of the EV) [93,98]. Recently, to solve this problem, Nakai et al. used the Ca2+-dependent Tim4 protein, which binds selectively to phosphatidylserine, a protein that is highly expressed on the exosome surface, to build an exosome isolation device that overcomes the limitation imposed by antibody capture [99].

### 3.4. FACSCanto and EV Sorting

Since flow cytometry would enable high-throughput, multi-parametric analysis and separation (sorting) of individual EVs depending on their surface features, it seems of interest from a translation point of view. This is the reason behind the multiple efforts invested to improve this method. Low refractive index and submicron size of EVs are significant disadvantages when employing this method. Particles smaller than 600 nm are below the detection limits on forward/side scattered (FSC/SSC) light detectors. This results in electrical noise and scattered light signals that overlap with the buffers [100,101].

Recently, de Rond et al. [102] described a new flow cytometry approach that can detect and isolate 100 nm EVs. This study focused on enhancing the forward scatter (FSC) and side scatter (SSC) sensitivity of the FACSCanto flow cytometer to detect single 100 nm EVs. This is a crucial aspect for improvement because of the small size and low refractive index of EVs, which typically place them below the detection limits of standard flow cytometers. The study systematically evaluated various adaptations to the optical configuration and fluidics of the flow cytometer. These improvements included changes to the obscuration bar shape, laser power, pinhole diameter, and sample stream width, among others. The goal was to improve the detection of scatter signals from EVs. The optimized FACSCanto was tested with both polystyrene beads and EVs isolated from human urine. The results showed that the optimized system could effectively measure the scatter signals from these particles, confirming its enhanced capacity to detect EVs. The study reported significant improvements in sensitivity, achieving estimated detection limits for EVs of 246 nm for FSC and 91 nm for SSC. This implies that with the right configurations, flow cytometry-based methods can constitute a powerful tool for isolating and analyzing EVs [102].

**Figure 5:**
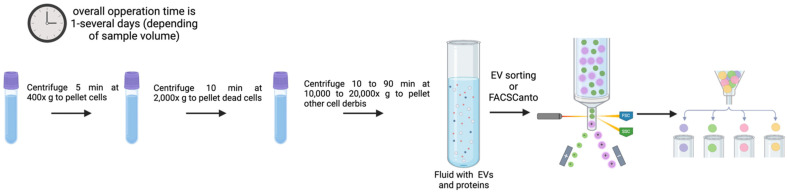
Schematic EV sorting procedure for EV isolation as described by Song et al. [103]. This procedure is specially used with cell culture medium and plasma samples. The working volume will condition the overall operation time [103] (created with Biorender).

Song et al. provided a thorough procedure (Figure 5) for isolating and purifying 50–200 nm small EV (sEV) using a flow cell sorter [103]. To achieve a stable side stream and a decent sorting rate, a 50 µm nozzle and 80 psi sheath fluid pressure were chosen. Standard-sized polystyrene microspheres were used to locate populations of 100, 200, and 300 nm particles. The sEV signal may be distinguished from the background noise with further adjustment of the voltage, gain, and FSC triggering threshold. A representative population of sEV can be obtained using FSC compared with SSC alone thanks to the panel of optimized sort settings. Numerous downstream research applications are possible with EVs isolated by the flow cytometry-based technology, which not only enables high-throughput analysis but also synchronous categorization or proteome analysis [103]. Despite the advantages of this technique, it is a high-cost procedure. Access to modified flow cytometry equipment is something that very few laboratories can afford [102,103].

### 3.5. UF

**Figure 6:**
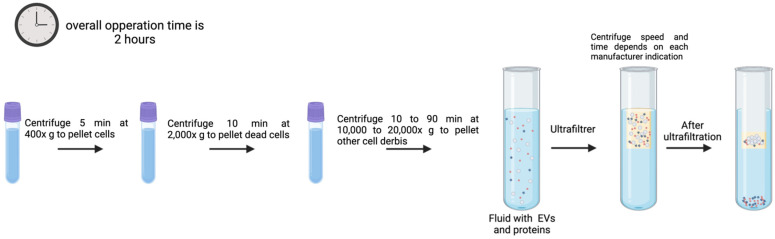
UF protocol overview for EV isolation. Working volumes and speed time differ between commercial filters distributors, but most commonly vary between 0.5 and 4 mL [67,104] (created with Biorender).

With a molecular weight cutoff (MWCO) value of 10, 50, and 100 kDa, UF is used as a fundamental step to extract EVs from large volumes of samples, into concentrated small volumes that can be used in further purification procedures or analysis (Figure 6) [104]. The idea behind this technique is that EVs will be purified with the use of membrane filters of pore size that limit the passage or flow of contaminants.

Using membrane filters with pore diameters of 0.22 and 0.45 μm, EVs are first separated from bigger pollutants such as cells, debris, and microparticles. Then, commercial membrane filters with molecular weight cutoffs ranging from 5 to 100 kDa, such as the Corning Disposable Bottle-Top Filter or the Acison Centrifugal Filter, are used to separate the soluble and aggregated proteins.

EVs are later separated using UF, which uses pressure to force sample fluid through membranes with pores smaller than 100 nm [67]. Membranes with nanoscale or greater pore widths can be used in additional procedures to filter out more unwanted particles. Although the procedure is faster than UC, the pressure used can result in vesicle loss due to membrane adhesion, vesicle fusion and damage from shear stress, and membrane blockage from particle aggregation, which might reduce EV yields and lengthen processing time [67,105]. Vesicle UF techniques include tandem filtration, centrifugal UF, tangential flow filtration, and sequential filtration [67,106].

Sequential filtration entails several rounds of filtration, each with a distinct molecular weight cutoff, whereas tandem filtration combines multiple filters in a single syringe. When the nanoporous membrane is rotated inside a tube, centrifugal force pushes the sample material through the membrane [107,108]. To prevent clogging and remove big particles from samples such as cells, intact organelles, apoptotic bodies, and protein aggregates, preliminary centrifugation or dead-end filtering at 0.22 μm prior to centrifugal UF is frequently used.

More recently, EVs have been isolated with greater yields using tangential flow filtration (TFF) [106]. Instead of applying pressure orthogonally, TFF filters samples in tangential disposition to the membrane [108]. EV loss may occur because of membrane damage and contamination by soluble components smaller than filter pores which are two drawbacks for this option. Additionally, some vesicles might be lost by absorption into the membrane at the crucial step of retrieving minute amounts of biological fluids. TFF can be scaled up to process larger volumes of fluid with improved consistency and with milder effects on the sample when compared to UC. However, TFF requires more enhanced processing time than other filtration methods.

Compared to UC-based isolation methods, UF is simpler, quicker, and requires less special equipment. UF can damage EVs by shear stress and induce particle aggregation, which might impact EV yield [67,105,106].

### 3.6. SEC

**Figure 7:**
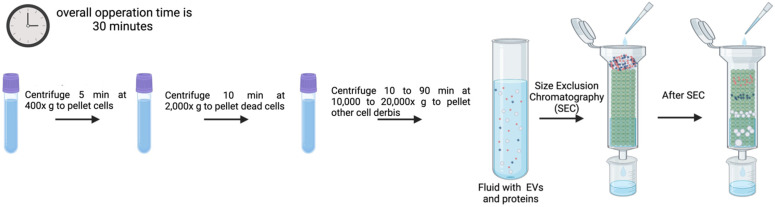
SEC summarized protocol for EV isolation as described by Böing et al. [110] for all types of biofluid samples. The working volume can be easily scaled up/down by changing column features, but it is important to know that the sample volume should not be greater than 1/20 to 1/15 of the column volume [114] (created with Biorender).

SEC or Gel Filtration is frequently used to separate biopolymers, such as proteins, polysaccharides, proteoglycans, etc., from fluids by allowing molecules with different hydrodynamic radius to be separated. This method can also be used to isolate EVs from blood plasma, protein complexes in urine, and lipoproteins [109,110,111,112,113]. Since vesicles travel with the fluid flow (Figure 7) under a slight differential pressure and, seldomly interacting with the stationary phase, EVs keep intact their integrity, preserve biological activity, and reflect abundance in origin during SEC [114,115]. Furthermore, biopolymer interaction and nonspecific contamination of EV preparations are reduced when high ionic strength buffers are used [114]. Additionally, chromatography is easily scalable, with longer columns improving peak resolution for particles that are close in size, while larger columns enable the analysis of more concentrated materials from larger volumes. However, it is important to mention that the number of components, the volumes analyzed, and the variation in diameter (hydrodynamic radius) of the particles to be separated impact separation efficiencies [114].

Several commercial column types have been developed to make the process of EV isolation by SEC easier and more reproducible. These include Sephacryl S-400 (Cytiva (formerly GE Healthcare), Uppsala, Sweden), Sepharose 2B (Cytiva (formerly GE Healthcare), Uppsala, Sweden), Sepharose CL-4B (Cytiva (formerly GE Healthcare), Uppsala, Sweden), Sepharose CL-2B (Cytiva (formerly GE Healthcare), Uppsala, Sweden), qEV Size Exclusion Columns (Izon Science Ltd., Christchurch, New Zealand), and Exo-spin Columns (Cell Guidance Systems, Cambridge, UK) [116,117,118,119]. When EV isolation efficiency is compared to commercial columns, it is shown that the resulting EV preparations differ in terms of both efficiency and albumin contamination level [116].

### 3.7. Microfluidics

There are different types of microfluidic devices developed to isolate EVs. The methodology described in 2015 by Dudani et al. [120] for the isolation and detection of EVs focuses on a microfluidic platform based on immunoaffinity capture of vesicles using a technique called Rapid Inertial Solution Exchange (RInSE). To perform this method, a microfluidic device is needed where fluid is introduced using PHD 2000 syringe pumps (Harvard Apparatus, Holliston, MA, USA) and Polyetheretherketone (PEEK) tubing (Spectrum Plastics, Alpharetta, GA, USA). This setup allows for precise control of fluid flow rates during the isolation process.

An example of this protocol to isolate exosomes begins with the preparation of 20 µm polystyrene beads that are coated with streptavidin and then incubated with biotinylated anti-human CD63 antibodies, a surface marker of exosomes, at 37 °C for 30 min for bead coating. After removal of unbound antibodies, coated beads are resuspended in tris-buffered saline (TBS). This ensures that the beads are ready for the isolation of EVs from the sample solution (culture media or blood samples) [120]. After the introduction of the anti-human CD63 antibody-coated beads into the microfluidic device, the fluid is pumped through the device with syringe pumps, allowing the selective capture of exosomes by the beads in the microchannels. These devices operate at higher flow rates than traditional methods, enhancing the volume of fluid processed and, in consequence, improving the efficiency of EV isolation. The inertial forces help to separate EVs from other particles in solution, enhancing the specificity of the isolation process. Once the EVs are captured on the beads, an elution buffer (IgG elution buffer) is added to release the EVs from the beads. This step is crucial for obtaining isolated exosomes in a solution that can be further analyzed by their content and features [120].

In particular, the signal detection-based microfluidic approach [120,121] is one of the most recent techniques for separating exosomes from low amounts of biological fluids by using optical, electrochemical, or magnetic signals to detect exosomes. Microfluidics devices enable quick, precise, and economical isolation of exosomes from other nanometer-sized particles [121,122]. Popular microfluidic-based technologies fully integrate size-based, immunoaffinity-based, and dynamic separation of vesicles. It seems worth mentioning the recently developed ExoTIC device to isolate exosomes from serum or other physiological fluids [87,121], which outperforms PEG precipitation and UC alternatives in terms of yields, purity, and efficiency [122].

Integrated systems, which consist of two or more devices constructed to function independently but in parallel, are known as microfluidic systems. Usually, a network of linked microchannels made up of one or more devices can handle smaller volumes of sample [123,124]. This feature makes microfluidic devices capable of accurately and specifically replicating intricate analytical processes at the microscale level. To promote fluid movement or expand the range of possible selection criteria, more specialized components might be added [125].

Other devices incorporate immunoaffinity-based microfluidic EV isolation techniques, which capture analytes using a general lateral flow [126]. This process involves coating the base of a microfluidic device, like the ExoChip (made of polydimethylsiloxane), with antibodies against EV surface markers that are frequently overexpressed, such as CD9, CD63, and CD81 for exosome subpopulations [127]. Still another example is represented by arrays of silicon nanowire micropillars that employ SEC to capture exosomes [128]. Exosomes are caught in openings of this system as the fluid is pushed through. The initial phase of this process is quick, but it may take up to 24 h for exosomes to be released from the pores, limiting its diagnostic adequacy in terms of clinical settings.

**Figure 8:**
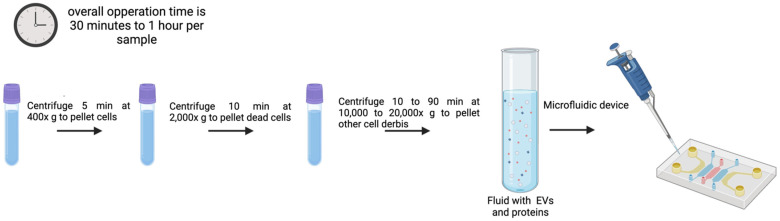
Overall procedure steps followed by microfluidic–based methods of EV isolation. Operation time is estimated to range from 30 min to 1 h per 0.2 mL sample. The working volume range spans from 1 picoliter to 200 mL [128,129,130,131,132] (created with Biorender).

EV separation by size has also been accomplished using a viscoelastic-based microfluidic platform [129,130,131]. Before being placed in viscoelastic buffer, samples are mixed with biocompatible, elastically responsive polymers of this platform. As the fluid passes through the devices, larger particles—such as cells, cell debris, and microvesicles from serum or cell culture medium with higher elastic pressures—are forced away from the EVs (Figure 8). Meng et al. describe one example of this subtype of microfluidic platform to allow for the continuous and label-free isolation of exosomes straight from cancer patient blood samples. At a sample volumetric flow rate of 200 μL/h, they found that the device effectively extracted EVs with a diameter of around 100 nm, demonstrating 97% purity and 87% recovery rate [131].

Acoustic microfluidic approaches represent another variant of microfluidic technology that can be used to separate exosomes based on their size [122,127]. In contrast to micropillar arrays, acoustic waves are milder on vesicles and require less contact. A flowing sample is used by interdigital transducers to create waves all over it. The particle size cutoff at the two separation channels is determined by the wave frequency. While the waste from one channel solely contains exosomes with a purity of around 98%, the residue from the other channel contains both big microvesicles and apoptotic bodies. Each sample takes around 25 min to run in this scenario, and EVs maintain their biological function because there is no physical interaction.

In addition to using acoustics, microfluidics can also use electrical waves to separate exosomes without the need for labels or physical touch [133]. Ion-based separation makes use of exosomes’ greater negative charge in comparison to other particles as the principle for its purification [134]. Mogi et al. developed a microfluidic device of this type containing two inlet and two exit channels. High and low voltage is used to separate positively and negatively charged particles [134]. Positively charged particles are attracted to the low-voltage channel in this device, whereas negatively charged particles are attracted to the high-voltage channel in each pair. A perpendicular ion channel in the middle creates an ion depletion zone that pushes uncharged particles into the channels and keeps them toward the center. This device, which is tuned for voltage and flow rate to improve exosome retention, has demonstrated notably greater yields than UC methods.

Microfluidics-based technology can isolate EVs from a reduced volume of sample. It enhances capture efficiency and specificity, paving the way for automated and high-throughput EV detection in clinical settings. Another strength of this isolation method is that EVs keep their function capacity unaltered after their isolation. However, costs are still high, and it is time consuming [131].

### 3.8. EV Isolation Methods Overview

The main EV isolation methods rely on different principles, leading to particular advantages and disadvantages that are sometimes difficult to compare. To help choose the isolation method that fits an experimental aim best, we have created a summary table (Table 1) with strengths and weaknesses for each of the methods depicted here.

### 3.9. EV Quantification and Characterization

Concentration and purity of isolated EVs is usually determined as a first step in many protocols. This may include EV quantification, particle size distribution, EV morphology, and characterization of EVs by molecular composition [31,97,135]. Another critical parameter to understand the stability and behavior of EVs is the zeta potential which describes EV electric properties, conditioning EV interactions with their extracellular environment, and their capacity to fuse [136,137]. The main methods to quantitate and characterize EVs are summarized below:

Western Blot analysis is used to semi-quantitatively assess EV presence in the sample [97,117,138]. There is no universal EV marker to characterize all EV subtypes. However, antibodies against tetraspanins CD63, CD9 or CD81, and anti-ALIX or anti-TSG101 are commonly used [116].

Specialized flow cytometry detects EVs with a minimum size of 50 nm [102,103]. Measurements include EV concentration, diameter, epitope abundance, and effective refractive index. Welsh et al. reported a specific protocol to perform single EV characterization using this method [31,139,140]. EV surface proteins have been extensively examined using bead-based flow cytometry, capturing EV with specific fluorescent reagents attached to identify bead-associated particles. Since the signal is derived from many particles that are attached to individual beads, variations in staining intensity are only semi-quantitative [139,140,141].

Mass spectrometry (MS), which measures mass-to-charge ratios, is frequently employed to identify EV-associated proteins for both targeted and discovery applications [142]. Time-of-Flight or Orbitrap MS platforms are used for untargeted proteomics, whereas triple quadrupole liquid chromatography (LC)-MS platforms are chosen for targeted analysis [119,142]. This method offers thorough and comprehensive information on protein composition and, thus, is suitable for biomarker identification [143,144,145,146,147].

Dynamic Light Scattering (DLS) or photon correlation spectroscopy is an optical technique that uses a laser beam to measure EV diffusion velocity and size [116,135]. The laser beam strikes the particle solution, and in the presence of particles (EVs), the light is dispersed and scattered [97,135]. A digital autocorrelator tracks the change in light scattering signal caused by the particles’ Brownian motion to obtain EV diffusion velocity and size. DLS can also measure the EVs’ zeta potential [116,142,145].

Nanoparticle Tracking Analysis (NTA) is commonly used to visualize and characterize EVs [31]. This technique gives real-time information on the size, size distribution, zeta potential, concentration, and/or markers on their surface (if using the fluorescent mode) [31,116,117]. The NTA technique also tracks the Brownian motion of nanoparticles in solution using a laser beam. It uses a highly sensitive camera to capture the scattered light from the nanoparticles in real-time. NTA analyzes the nanoparticle trajectories in different frames each at a time within a certain period, so the diffusion coefficient of each nanoparticle is calculated according to its motion. Its hydrodynamic diameter is subsequently calculated using the Stokes–Einstein equation [135,142]. There are two types of signal detection, one in which the signal from the scattered lights of the EVs is detected and another in which the fluorescent emission from the fluorescently labeled EVs is measured. This fluorescent mode allows us to distinguish the different subtypes of EVs and gives more accurate results by discarding the signal coming from contaminating non-EV particles [135,145,148]. However, EV purity, measurement temperature, sample dilution, and other factors may impact NTA results. Equipment manipulation, calibration, and optimization of settings are also important factors to obtain reliable results [145,148].

Atomic Force Microscopy (AFM) can image individual EVs and co-isolated nanoparticles without the need for labels or stains. Its detection system consists of a laser and a position-sensitive detector (a photodiode) that records the cantilever (a flat spring with a sharp point sensor) deflection caused by the forces acting on its tip when the sample is nearby [135,142]. The capacity of the AFM technology to measure samples in their natural state with the least amount of sample preparation is one of its key advantages [149,150,151]. EV size distribution and ultrastructural features, as well as the presence and relative concentrations of pollutants, can be obtained via AFM morphometry. Furthermore, AFM is among the few methods that can measure nanomechanical characteristics of a single vesicle [135]. With AFM technology, size distribution and diameter of EVs can be determined. There is software to produce true 3-D imaging of EV surface topography with extremely high resolution [149,150,151].

Fluorescence microscopy techniques are used for single-vesicle localization and live cell imaging. These methods, which include confocal microscopy, light-sheet microscopy, and Total Internal Reflection Microscopy (TIRF-M), are used to study EV composition as well as cell–EV interactions such as EV release and uptake [145].

Electron microscopy techniques are straightforward methods to determine the size and shape of individual EVs [97,116]. Three categories—scanning electron microscopy (SEM), transmission electron microscopy (TEM), and cryo-transmission electron microscopy (cryo-TEM)—are commonly used to document size and shape of a single EV’s particles [116,135].

SEM works by using a concentrated electron beam to scan a sample, producing high-resolution images depending on how the electrons interact with the sample’s surface. This technique overcomes the difficulties caused by the tiny size and variety of EVs enabling comprehensive morphological and size-based profiling of EVs [138,142].

TEM is one of the most widespread techniques for visualizing EVs, providing EVs’ size and morphology details [142]. It uses a mechanism based on electron beams that change their scattering angle after striking EVs [31,116]. TEM gives a better resolution than SEM, holding the capacity to analyze single EVs [97,135].

Cryo-TEM was developed to preserve the authenticity and integrity of the sample to the maximum extent possible while reducing protocol’s complexity [138,142]. In addition, the cryo-TEM approach enables internal EV examination [116,135].

Tunable Resistive Pulse Sensing (TRPS) is another technique that analyzes EV size distribution, concentration, and zeta potential [135]. It uses the Coulter principle to attain EV physical characteristics operating by measuring electrical resistance changes as non-conductive vesicles pass through a pore tunnel, a change referred to as resistive pulse [152]. By analyzing these resistive pulses, the size of EVs can be determined [97,152,153].

Interference Reflectance Imaging Sensor (IRIS). Here, particles are captured by affinity agents (such as aptamers, peptides, or antibodies) onto a multiplexed array of micron-sized dots and combined with interferometric imaging/fluorescence imaging [31]. The size and quantity of particles are recorded by using interference patterns from scattered light with the IRIS mode. The refractive index, which varies among EV populations, determines how interference is converted to nominal size. Fluorescence mode detects particles in one or more color channels after being labeled with fluorescent probes [31,142].

Fluorescence Super-Resolution Microscopy prevents neighboring molecules from emitting simultaneously to avoid fluorescence emission events close to light diffraction limits [97]. Two primary methods are available: Single-Molecule Localization Microscopy (SMLM) techniques, like STORM and PALM, which temporally regulate stochastic activation of single fluorophores; and Stimulated Emission Depletion (STED)-based methods which spatially regulate the activation of fluorophore assemblies using a synchronized two-laser system on a phase plate to give high-resolution images [97,147,153,154,155,156]. STED and SMLM’s nanoscale resolution makes them ideal for identifying and describing distinct EVs and their proteins, membranes, and cargo-like DNA fragments or miRNAs [153,154,155,156].

Raman Spectroscopy (RS) is a type of light scattering technology that identifies and examines the vibrational modes of molecules by detecting inelastic scattered light. It offers molecular fingerprints of samples and permits the tracking of modifications affecting molecular bond structures. It is possible to determine the chemical composition of EVs using RS-based techniques [141]. The use of strong spectroscopic methods for enhancing the RS signals, surface-enhanced Raman scattering (SERS) and Tip-Enhanced Raman Scattering (TERS), allow the analysis of EVs at the single-molecule level [157,158,159].

Fourier-Transform Infrared (FT-IR) spectroscopy is a powerful non-invasive and label-free analytical technique used to characterize the biochemical properties of EVs by measuring infrared radiation absorption to identify molecular functional groups [160,161]. This technique provides insights into the composition, structure, and interactions of biological samples previously dried on a solid surface, such as a silicon plate [160,161,162].

Fluorescence Correlation Spectroscopy (FCS) determines the size of fluorescently labeled EVs by analyzing changes in fluorescence intensity. The ability of FCS to identify a single, fluorescently labeled molecule is its primary benefit over DLS [163,164].

Micro-Computed Tomography (micro-CT), another name for X-ray microscopy, is a promising technique for determining the size and shape of EVs in their physiological state. Micro-CT is a relatively recently developed imaging technique that can examine thick samples at a resolution halfway between that of electron and optical microscopy [135,165,166].

It is possible to modify the popular Enzyme-Linked Immunosorbent Assay (ELISA) to detect EVs, particularly exosomes [117,135]. Exosomes from various biological materials, such as cell culture supernatants and human body fluids are captured by pan-exosome or cell/tissue-specific antibody pre-coated surfaces [167].

Lastly, Yoshioka et al. reported an ultra-sensitive technique known as ExoScreen for the identification of tumor-derived exosomes in the serum of patients with colorectal cancer [168,169]. The technique is based on the FRET (Fluorescence Resonance Energy Transfer) principle to detect pairs of markers in the surface of EVs. For example, donor phtalocyanine-laden beads coated with streptavidin to capture pan-exosome biotinylated antibodies, such as anti-CD63, are exposed to acceptor beads conjugated to a secondary antibody that recognizes a tissue/cell specific epitope on exosomes, and then stimulated by a laser at 680 nm, which transforms endogenous diatomic oxygen into singlet oxygen. This causes the acceptor beads (loaded with thioxene) to exhibit an enhanced fluorescence signal at 615 nm only when both surface markers are present at a distance 200 nm or less [168,169].

## 4. Biogenesis and Function of microRNAs

Mature miRNAs play significant roles in gene regulation, affecting important biological processes such as development, differentiation, apoptosis, and immune responses [170]. As previously mentioned, dysregulated miRNA expression is observed with disease. Examples include cardiovascular disorders [171], cancer [172], or neurodegenerative disease [173], as previously mentioned.

### 4.1. microRNA Biogenesis

miRNA biogenesis begins with the transcription of a long primary transcript called pri-miRNA typically transcribed by RNA polymerase II, which contains a hairpin-like structure [174]. These pri-miRNA are processed in the nucleus by a micro-complex, primarily formed by the Drosha ribonuclease and the double-stranded RNA-binding protein DGCR8 (DiGeorge Syndrome Critical Region 8) to generate a shorter, hairpin-structured pre-miRNA (Figure 1) [175]. The pre-miRNA is then exported from the nucleus to the cytoplasm via a protein called Exportin-5, which recognizes 2-nucleotide overhangs at the ends of the pre-miRNA structure [176]. Once in the cytoplasm, the pre-miRNA is processed further by the Dicer ribonuclease enzyme, which cleaves the loop of the pre-miRNA to produce a miRNA duplex that consists of a guide strand and a passenger strand. The guide strand is incorporated into the RNA-induced silencing complex (RISC), whereas the passenger strand is typically degraded [177,178]. The mature miRNA incorporated into the RISC guides the complex to complementary messenger RNA (mRNA) targets. The interaction between the miRNA and its target mRNA often leads to translational repression or mRNA degradation, thus regulating gene expression (Figure 1) [179,180,181].

Some alternative mechanisms and processes contribute to miRNA regulation and function; for instance, some studies indicate that Dicer can be processed into different isoforms or interact with other RNA-binding proteins (RBPs), which can alter the processing of pre-miRNAs [182]. A recent study has shown that certain miRNAs may bypass Dicer processing. These Dicer-independent pathways involve alternative RNA-processing enzymes like Argonaute 2 (Ago2), which directly cleaves precursor miRNA molecules into mature miRNAs [183].

miRNA biogenesis can be modulated by tissue-specific factors or external stimuli such as stress, disease, or changes in the environment. This means that different tissues may have different sets of miRNAs, or certain signaling pathways may regulate the processing of specific miRNAs [184]. RBPs can also regulate miRNA biogenesis by interacting with pri-miRNAs or pre-miRNAs [185].

Some pri-miRNAs may undergo alternative splicing, generating multiple isoforms of pre-miRNAs or even different mature miRNAs from a single miRNA gene. This allows for finely tuned regulation of miRNA function, particularly in complex biological processes like development, cell differentiation, and responses to stress [186].

A recent publication suggests that long non-coding RNAs (lncRNAs) can be processed to produce functional miRNAs. These lncRNAs may contain embedded miRNA genes or undergo complex splicing events that yield miRNA-like molecules, contributing to miRNA biogenesis from alternative RNA species [187].

The exosome complex, known for its role in RNA degradation, can also participate in the processing of certain miRNAs. This suggests that there could be alternative pathways for miRNA maturation that do not involve the canonical Drosha/Dicer pathway [53,188].

### 4.2. microRNA Function

miRNAs primarily function as post-transcriptional regulators of gene expression [189,190]. miRNAs are implicated in various biological processes, including development, differentiation, and cell cycle control [144,145,146,147], also participating in pathological processes [53,54].

miRNAs regulate gene expression by binding to complementary sequences within the 3′ untranslated region (3′ UTR) of target mRNAs [189], thus recruiting the RISC to degrade the mRNA or inhibit its translation. This regulatory capacity allows miRNAs to control key cellular processes, such as proliferation, differentiation, apoptosis, immune response, and metabolism [190,191,192,193].

In a disease context, miRNAs may play different roles. For example, in cancer, miRNAs often act as either oncogenes (oncomiRs) or tumor suppressors; e.g., miR-21 is an oncomiR frequently upregulated in cancers, promoting tumor growth and metastasis by targeting tumor suppressor genes such as PTEN and PDCD4. Conversely, the let-7 family of miRNAs functions as tumor suppressors by inhibiting oncogenic pathways, including those mediated by RAS and MYC [194]. Dysregulation of miRNAs is implicated in a broad spectrum of diseases beyond cancer, including cardiovascular disease, neurodegenerative disorders, and immune dysregulation-associated pathologies [195].

### 4.3. microRNAs in Therapy

miRNAs have emerged as promising therapeutic targets due to their critical roles in disease mechanisms and their simplicity and stability [196]. Some strategies to modulate miRNA activity include the following:

Antisense Oligonucleotides (ASOs): Synthetic nucleotides known as ASOs could selectively attach to target mRNAs or miRNAs and disrupt or block their activity. In the context of cancer, this strategy could work to restore tumor suppressor expression. ASOs can also be chemically modified to further improve stability and specificity for improved results. By specifically targeting oncogenic miRNAs that are important in carcinogenesis, such as miRNA-23a and miRNA-106b, ASOs have shown to significantly reduce their activity [197]. Because of their capacity to specifically target miRNAs and their engineered stability, ASO treatments offer promising therapeutic options for novel cancer treatments [198].

AntagomiRs: AntagomiRs are a specific type of ASO designed to inhibit miRNAs. Antagomir therapy is a promising approach for the modulation of miRNA activity in various medical conditions, including cardiovascular disease [199], neurological disorders [200], and cancer [201]. This therapeutic strategy involves the use of chemically modified oligonucleotides designed to inhibit specific miRNAs, thereby restoring normal gene expression and cellular function. AntagomiR-21 has shown to improve cardiac function post-myocardial infarction by reducing myocardial fibrosis and apoptosis, leading to enhanced ejection fraction and decreased left ventricular end-diastolic diameter [199]. In addition, in a study performed by Huang et al. on ischemic stroke models, miR-15a/16-1 antagomiR significantly improved neurobehavioral outcomes and reduced infarct volume [200].

Small-Molecule Inhibitors: Small-molecule inhibitors can block the pathological process by interfering with elements involved in miRNA biogenesis pathways. These inhibitors seek to restore normal gene expression patterns through different mechanisms, for instance, limiting tumor growth by suppressing oncogenic miRNA activity [202]. Anti-miR-122 therapy is being explored as a potential treatment for hepatitis C virus (HCV) infection and related liver diseases due to the critical role of microRNA-122 (miR-122) in HCV replication. The therapeutic approach aims to inhibit miR-122 activity, thereby reducing HCV replication and potentially improving patient outcomes [203,204]. These inhibitors work by transcriptionally inhibiting miR-122 expression, showcasing a novel therapeutic strategy against HCV [205]. The use of anti-miR-122 therapy could lead to significant advancements in HCV treatment, particularly for patients with chronic infections where current antiviral therapies may be insufficient [203].

Ongoing clinical trials are expanding the use of miRNA-based therapeutic approaches for the treatment of genetic disorders, showcasing their versatility and wide potential [206,207]. In the context of this focused review, it seems relevant to highlight that delivery vehicles such as nanoparticles and EVs are being explored to enhance the stability and specificity of miRNA-based therapeutics [10,201].

### 4.4. microRNAs in Diagnosis

miRNAs’ diagnostic value has been amply recognized due to their stability in biological fluids, disease-specific expression patterns, and non-invasive accessibility. Aberrant miRNA expression profiles have already been identified for a variety of diseases (Table 2), allowing early detection, progression of disease, and prediction of treatment response [3,9].

Despite promising miRNA applications [218,219,220], clinical implementation of miRNA diagnostics faces the challenge of high variability in circulating miRNA expression levels, potentially making results hard to interpret. An aspect further made difficult by the lack of standardized protocols [221] and the limited sensitivity and specificity of current methods when evaluating heterogeneous samples [222].

In the initial experimental discovery phase of miRNA biomarkers, high-throughput -omic screens, such as microarrays, followed by validation of the most promising candidates by quantitative polymerase chain reaction (qPCR) measurements are popularly used [223].

## 5. Methods to Assess miRNA Levels

The diagnostic use of miRNAs relies on their accurate detection and reproducible quantification in body fluids such as blood, urine, and saliva. Common methodologies include the following:

### 5.1. Quantitative Real-Time PCR (qRT-PCR)

One of the most popular and sensitive methods for measuring miRNA expression levels is qRT-PCR. Since miRNAs are short (~19–25 nucleotides) [24], they cannot be directly retrotranscribed like mRNAs. To overcome their lack of polyA tail to anchor oligo-dT primers, the process includes a polyadenylation reaction prior to their reverse transcription (RT). qRT-PCR options include TaqMan-based miRNA TaqMan test (Life Technologies, Carlsbad, CA, USA), SYBR green-based miScript (Qiagen, Hilden, Germany), and SYBR green-based miRCURY LNA (Exiqon, Vedbæk, Denmark) [224]. These popular methods have evolved into qRT-PCR arrays that can profile huge sets of circulating miRNAs at once, such as the TaqMan array miRNA 384 Cards (Thermo Fisher Scientific, Waltham, MA, USA), Smart Chip PCR (Takara Bio Inc., Kusatsu, Japan), and bespoke miScript miRNA PCR array (Qiagen, Hilden, Germany) [225].

qRT-PCR is a sensitive technique for measuring miRNA levels [225,226]. An amplification curve is produced using real-time monitoring of fluorescence linked to the amplification of cDNA obtained from miRNA species [227]. Numerous software programs are available for analyzing qPCR curves, yielding quantification [228].

Despite being a sensitive technique, problems may occur when working with target levels that are close to qPCR detection limits, as in the case of many circulating miRNAs. This results in missing data, which are processed and interpreted differently by several studies. There is currently no agreement on how to deal with missing data in qPCR experiments in a statistically solid way [224,225]. Therefore, it can be said that qRT-PCR is a highly sensitive and specific method to detect miRNAs, but it is limited when the target abundance is close to the qRT-PCR detection limits [229].

To specifically approach miRNA detection improvement, *stem-loop priming* was developed. Stem-loop priming is a widely used technique for the reverse transcription of miRNAs, offering high specificity and efficiency in miRNA quantification. Unlike conventional linear primers, stem-loop primers are designed to anneal to the 3′ end of the miRNA, forming a hairpin structure that enhances the specificity of reverse transcription and prevents the amplification of precursor miRNAs. This method enables selective detection of mature miRNAs while reducing background signals from primary and precursor forms. Stem-loop priming also improves cDNA stability and facilitates efficient amplification [230].

### 5.2. Microarray Analysis

As mentioned, an established -omic method for examining unbiased microRNA expression patterns is microarray analysis. Microarrays use probes complementary to known miRNAs immobilized on a solid surface. Upon labeling, miRNA samples are hybridized to these probes, with the resulting fluorescence signals revealing their presence and relative abundance in the sample [231,232,233]. The ability to concurrently identify several miRNAs at once and detect them across body fluids is one of the many clear benefits that microarray analysis offers when it comes to miRNA biomarker identification. It constitutes a noninvasive and practical diagnostic technique, suitable for comparative expression profiling, despite holding lower sensitivity as compared to qRT-PCR. An additional limitation is that target probes can yield cross-hybridization, leading to the detection of false positives [231].

### 5.3. Next-Generation Sequencing (NGS)

By contrast to microarrays, NGS can render the discovery of novel miRNAs [234]. It involves converting miRNA into cDNA libraries, which are then sequenced to determine the exact nucleotide sequences and quantities of miRNAs present in a sample. With the help of NGS, millions of DNA fragments can be sequenced quickly at once, enabling precise microRNA detection and characterization across their whole sequence [234].

Background noise and cross-hybridization are issues that NGS technologies do not face when compared to other high-throughput techniques like microarrays. Other noteworthy benefits of the NGS include the ability to produce thorough and conclusive results of all miRNAs even in samples with low content such as plasma or serum [234], and it does not require previous knowledge of target miRNAs [225,235]. However, it is not free of limitations, with the main being its high cost and the requirement of time-consuming computational infrastructure for the analysis and interpretation of data [236].

### 5.4. In Situ Hybridization

The expression and location of molecules within a cell, tissue, or embryo can be detected using the traditional in situ hybridization (ISH) technique. ISH methods were originally applied to the detection of miRNA in 2006 [237]. They used tagged complementary nucleic acid probes to identify single-stranded DNA or RNA in tissue slices or fixed cells [238].

ISH’s main benefit over other miRNA detection techniques is its capacity to track both cellular and subcellular distributions and identify the spatiotemporal expression profile of miRNAs. This technique is useful to clarify the biological function of miRNAs and their potential pathological participation in a variety of disorders.

ISH is currently the only method for miRNA profiling that can identify the native localization of miRNA at the single-cell level, inside tissues, or in cell compartments [239]. Single-molecule RNA FISH (smRNA-FISH) approaches have been made possible by recent developments in a variety of signal amplification and super-resolution imaging techniques.

ISH, however, has low throughput that limits its applications, but it can identify many miRNAs per reaction [240]. FISH technology has advanced significantly with the development of new labeling techniques and the launch of high-resolution imaging systems for precise mapping of intra-nuclear genomic architecture and single-cell, single-molecule profiling of cytoplasmic RNA transcription, being used in the clinic for genetic diagnostics [241,242,243].

### 5.5. Northern Blotting

Northern Blot (NB) for the analysis of miRNA levels is an easily accessible technology. It is carried out by size-separating RNAs species in a sample using denaturing gel electrophoresis, transferring and cross-linking it to a membrane, and then hybridizing it with a labeled nucleic acid probe complementary to the target RNA. Individual miRNA expression levels can be examined using this classical method [244]. The benefit of NB analysis is that it can identify both mature and miRNA precursors at the same time. However, this method is costly, time-consuming, and requires labeling.

Quantitative analysis does not usually employ NB. It is regarded as semi-quantitative and offers data on the relative levels of RNA expression in a sample or between samples [245,246]. Both radioactive and non-radioactive probes can be employed for detection, and labeling techniques such as uniform probe labeling and end labeling are acceptable [247,248]. The most often used radioactive labeling method is ^32^P because of its high sensitivity. However, a major drawback is the short half-life of the probe, the health risk for the operator that needs to comply with safety precautions, and specialized training related to the proper use of radioisotopes [248].

### 5.6. Biosensors

Biosensors for the detection of microRNA have gained significant attention due to their potential for early disease diagnosis and monitoring in a clinical setting [217]. Recent advancements in biosensor technology have focused on enhancing sensitivity, specificity, and dynamic range, making them suitable for clinical applications [249]. Different types of biosensors have been developed as follows:

Nanochannel Biosensors: Solid-state nanochannel biosensors use allosteric DNA probes to achieve a programmable dynamic range for miRNA detection. By employing tri-block DNA architectures, these biosensors can adjust binding affinities, achieving a dynamic range of up to 10,900-fold which enhances their versatility in various applications [249].

Electrochemical Biosensors: Electrochemical biosensors have emerged as effective tools for detecting miRNAs, particularly in cancer diagnostics. The principle of electrochemical biosensors is fundamentally based on the interaction between biological elements and electrochemical transducers, which convert biochemical signals into measurable electrical signals. These biosensors use various enzymes and biomolecules to achieve high sensitivity and specificity in detecting analytes [232]. They are characterized by simplicity, sensitivity, and low cost, making them ideal for point-of-care applications. For instance, a novel electrochemical biosensor achieved a detection limit of 2.5 fM for miRNA-182-5p, demonstrating high sensitivity in complex samples [231,232].

Surface-enhanced Raman scattering (SERS) Biosensor: When molecules are bonded to the “nanostructure” of substrates, Raman signals can be amplified by surface-enhanced Raman scattering. Pyridine adsorbed on electrochemically rough silver electrodes provides the source for this phenomenon [250]. In 1979, pyridine’s increased signal was also detected in a silver and gold colloidal solution [251]. In line with the electromagnetic mechanism (EM), it has been proved that SERS is more of a “nanostructure effect” than a “surface effect”, with the critical role that surface plasmon resonance plays in SERS having been amply illustrated [18]. The potential distribution observed in an electrochemical environment, which correlates to the chemical enhancement mechanism (CM) model, can be used to show the critical function of charge transferred between a molecule and a substrate in SERS, which makes it suitable to detect miRNA particles [252,253]. Electromagnetic field and molecular polarization enhancement are the two models that these two mechanisms correlate to. While the latter focuses on the modification of the molecular electronic structure during the adsorption process, resulting in resonant Raman scattering, the former concentrates on enhanced electromagnetic fields on metal surfaces with an appropriate shape [254,255].

Surface Plasmon Resonance Aptasensors: The biotin-streptavidin dual-mode phase imaging surface plasmon resonance (PI-SPR) aptasensor represents another innovative approach, significantly improving detection limits and reducing nonspecific adsorption. This method allows for rapid, simultaneous detection of multiple miRNA markers, enhancing its utility in clinical settings [256].

Biosensor techniques can provide rapid and real-time miRNA detection and could be suitable for portable diagnostics. However, they require careful design and optimization, and the isolation efficacy can be affected by biological sample complexity [220].

### 5.7. Digital Droplet PCR (ddPCR)

ddPCR partitions a miRNA-containing sample into thousands of nanoliter-sized droplets. Each droplet undergoes individual PCR amplification, and fluorescence signals are measured to determine miRNA copy numbers without the need for a standard curve. Unlike qRT-PCR, ddPCR provides direct miRNA copy number measurements without the requirement of reference genes. It is capable of detecting low-abundance miRNAs, even in challenging samples like EVs [257,258], or in complex biological samples, such as plasma and serum. ddPCR is more expensive than qRT-PCR due to specialized reagents and equipment. It can analyze only a few miRNAs at a time compared to NGS, and the sample partitioning must be optimized for accurate quantification [257,258].

### 5.8. NanoString

NanoString technology or nCounter offers a highly sensitive and robust platform for miRNA detection, enabling precise quantification without the need for reverse transcription or amplification [259,260]. This digital hybridization-based approach uses fluorescent barcoded probes that directly bind to target miRNAs [259,260]. This allows the multiplexed detection of hundreds of miRNAs in a single assay. Unlike qPCR and NGS, NanoString provides direct and absolute miRNA quantification. Additionally, it provides a highly specific and sensitive option to detect low abundant miRNAs. It is reproducible and easy to use, making this technique a powerful tool for biomarker discovery and detection [259,260].

### 5.9. Overview of Methods to Quantify miRNA Levels

All miRNA detection methods described use different principles for their detection and therefore present different potential advantages and disadvantages which need to be evaluated according to specific goals. Table 3 provides a summary of each of the methods presented together with their particular strengths and weaknesses.

To further illustrate the potential of miRNA detection in diagnostics, Table 2 compiles some examples of circulating miRNAs and their link to a particular disease. The detection method used in each study is also detailed to further document resources for methods to quantify miRNAs.

## 6. microRNAs Encapsulated in Extracellular Vesicle in Diagnosis and Treatment

Encapsulated miRNAs, primarily found within EVs, offer a highly stable and biologically relevant source of disease biomarkers [1]. These vesicles protect miRNAs from RNase-mediated degradation and facilitate their transport between cells located at very large distances within an organism, contributing to intercellular communication in physiological and pathological pathways [8,9]. The selective encapsulation of miRNAs into EVs is often influenced by disease-specific mechanisms, leading to distinct expression signatures that can serve as diagnostic and prognostic indicators [5]. The different advancements in EV isolation and miRNA detection methods explained in previous sections highlight their potential in the context of precision medicine, enabling non-invasive detection and monitoring of disease. Some examples of EV-derived miRNAs identified as disease biomarkers are listed in Table 4, together with the method used for their identification.

Maintaining the stability and integrity of miRNA-enriched EVs during storage is essential for their study as biomarkers in clinical diagnostics. Preservation of miRNAs within EVs is greatly influenced by several variables, including temperature, time, and freeze–thaw cycles. It has been reported that miRNAs encapsulated in EVs present in serum can be stored for up to three months at −20 °C; however, higher temperatures accelerate the degradation of miRNAs [263,264]. In a longitudinal study carried out by Andreu et al., it was found that after 6–8 years miRNA levels stayed unaffected, while samples frozen for over 13–14 years had reduced miRNA contents [62]. According to Akers et al., EV-encapsulated miRNA levels from CSF were unaltered after being kept at room temperature for seven days [265].

**Table 4:** Examples of encapsulated miRNAs in EVs identified as disease biomarkers. The isolation and detection methodology used in the identification are included.

Biomarker	Associated Disease	Isolation and Detection Methodology	Reference
miR-21, miR-126, miR-146a	COVID-19	UC for EV isolation; qRT-PCR for miRNA detection	[266]
miR-21, miR-155	Lung Cancer	UC for EV isolation; qRT-PCR for miRNA detection	[29]
miR-122, miR-192	Hepatocellular Carcinoma	UC for EV isolation; NGS for miRNA detection	[267]
miR-29a, miR-181b	Alzheimer’s Disease	UC for EV isolation; Surface-Enhanced Raman Scattering (SERS) for miRNA detection	[268]
miR-21, miR-141	Prostate Cancer	UC for EV isolation; qRT-PCR for miRNA detection	[269]
miR-21, miR-1246	Esophageal Squamous Cell Carcinoma	Glycosylated EV capture strategy; qRT-PCR for miRNA detection	[270]
miR-155, miR-210	Diffuse Large B-Cell Lymphoma	UC for EV isolation; qRT-PCR for miRNA detection	[271]
miR-21, miR-29a	Colorectal Cancer	UC for EV isolation; qRT-PCR for miRNA detection	[272]
miR-1246, miR-4644	Pancreatic Cancer	UC for EV isolation; qRT-PCR for miRNA detection	[273]
miR-21, miR-221	Glioblastoma	UC for EV isolation; qRT-PCR for miRNA detection	[274]

The ability of EVs to protect miRNAs from enzymatic degradation enhances their stability and bioavailability, making this route attractive for therapeutic delivery [10]. Moreover, engineered EVs can be designed to carry specific miRNAs to particular destinations, enabling targeted modulation of disease pathways [10,201]. EV-derived miRNAs and other EV cargos show the modulation of diverse cellular activities in different tissues and potential therapeutic efficacy in disease models [10]. The EV-derived miRNAs of MSC-derived EVs were suggested as the main bioactive compartment for their therapeutic effects [10]. An additional example is provided by the finding that encapsulated miR-150-3p increases osteoblast development and proliferation in osteoporosis [275]. miRNAs enclosed in MSC-derived EVs also showed possible modulation in cancer models. MiR-15a was shown to slow the evolution of carcinomas by blocking spalt-like transcription factor 4, while EV-derived miR-139-5p seems to prevent bladder carcinogenesis by targeting the polycomb repressor complex 1 [276]. In addition, tumor development and angiogenesis were effectively inhibited by EVs loaded with miR-497 [277].

However, additional work is needed for the validation of many of the findings and to standardize protocols before EV-encapsulated miRNAs can be implemented as treatments.

### Clinical Status

The clinical landscape for EVs as biomarkers is rapidly evolving, with significant advancements and ongoing research. A total of 49 clinical trials (11 completed) studying EVs as biomarkers are currently registered on clinicaltrials.gov. In addition, there are 287 clinical trials registered that explore the potential of microRNAs as biomarkers, with 91 of them reported as completed.

There has also been a significant rise in the number of clinical trials assessing the therapeutic potential of EVs, with over 106 trials registered for various conditions, including Crohn’s disease, ischemic stroke, type-I diabetes, and COVID-19. Among these trials, 20 are registered as completed, but published results are limited [278]. This indicates a need for more comprehensive data to validate the efficacy and safety of EV-based therapies. Early clinical evidence suggests that EVs, particularly those derived from mesenchymal stem cells (MSCs), are well tolerated in patients [278,279]. Readers are directed to the recent systematic review by Mizenko et al. for detailed updated information on EV clinical trials [280].

EVs have shown biocompatibility in early clinical studies, indicating their potential as allogeneic (“over-the-counter”) therapeutic options. However, there is still a lack of robust clinical evidence to support EVs’ widespread use in clinical settings. Initial clinical trials have demonstrated the safety of EVs, such as autologous dendritic cell (DC)-derived EVs to treat melanoma and non-small cell lung cancer (NSCLC). While these studies reported acceptable tolerability, they did not achieve significant therapeutic outcomes. Although studies suggest that EVs may stimulate both innate and adaptive immune responses, which could enhance anti-tumor effects [281,282,283], the mechanisms by which EVs exert their therapeutic effects are not fully understood.

An observational study by Motawi et al. compared the expression levels of specific miRNA signatures (miRNA-136, miRNA-494, miRNA-495) present in EVs in patients with pre-eclampsia with respect to normal pregnancies, finding that they were overexpressed in pre-eclampsia. An aspect that made the authors propose these EV-encapsulated miRNAs as early biomarkers for identifying pregnancies at-risk of suffering from pre-eclampsia (NCT03562715) [284,285].

A phase 2 interventional trial completed in 2019 used DNA from bronchoalveolar fluid EVs to assess Olmutinib mutagenic potential in non-small cell lung cancer (NSCLC) (NCT03228277) [286].

The EPI (ExoDx Prostate IntelliScore) assay, developed by Exosome Diagnostics, is a non-invasive urine test that identifies EV RNA biomarker signatures linked to high-grade prostate cancer. Clinical trials have shown its effectiveness in predicting prostate cancer risk and identifying patients who may avoid invasive biopsies (NCT02702856, NCT03031418, NCT04720599, NCT03235687). The EPI test was commercialized in 2016 and included in the National Comprehensive Cancer Network’s guidelines in 2019. It has received FDA breakthrough device designation and CE approval for clinical use in Europe [287,288,289].

The release and cargo of EVs can vary significantly among patients due to several factors including genetics, lifestyle, sex, and age. For instance, research indicates that EV release decreases while internalization by B cells increases with aging [290]. This variability poses challenges for developing personalized EV-based diagnostics. EV-based diagnostics cannot be generalized as a “one size fits all” solution. The unique biological context of each patient necessitates tailored approaches, which complicates the development of standardized diagnostic tests [291]. These challenges highlight the complexities involved in translating EV-based diagnostics from research to clinical practice, emphasizing the need for rigorous validation and standardization of methods to study EVs and their contents.

The clinical translation of EV-based diagnostics faces several significant challenges that hinder their widespread adoption. Although there are several CLIA (Clinical Laboratory Improvement Amendments)-certified EV-based diagnostic assays for clinical use, none have yet received FDA approval [292,293]. This limits the application of EV-based diagnostics to individually approved laboratories, restricting broader access to these diagnostic tools. Many identified RNA and protein signatures associated with EVs have not been rigorously validated [291]. This lack of validation raises concerns about the reliability and reproducibility of these biomarkers in clinical settings. In fact, different studies report varying conditions, such as particle counts or protein quantities, complicating inter-study comparisons and the establishment of universal criteria [169,278]. In consequence, some products in late-stage clinical trials still require widespread approval from agencies like the FDA and EMA [292,293]. Ongoing research strives to enhance the consistency and quality of EV preparations to facilitate their clinical use [278,292,293].

EVs are also being explored as drug delivery systems due to their inherent targeting properties, which may outperform synthetic nanoparticles. They can carry various therapeutic agents, including anti-tumor drugs and RNA therapeutics, making them versatile carriers [294,295,296].

To date, however, there has been no therapeutic EV product with approval from regulatory agencies like the FDA, which poses a significant barrier to their clinical translation [278,292]. While early trials report a lack of adverse events, there are concerns about potential toxicity and safety, especially with some EV sources and modification methods. The risk of tumorigenicity from cancer cell-derived EVs is of particular concern [169,297], as well as off-target effects, toxicity, and manufacturing challenges [292,298].

## 7. Discussion

EVs have gained increasing attention as potential biomarkers due to their ability to carry microRNAs that reflect the physiological and pathological states of their tissue of origin. Various isolation techniques, including UC, precipitation, immunoaffinity, sorting, UF, SEC, and microfluidics, have been employed to obtain EVs from biological samples. However, each method presents inherent limitations related to yield, purity, and processing time [103,104].

One of the primary challenges in EV research is the heterogeneity of vesicles and the diversity of isolation protocols, which can lead to inconsistencies in downstream analyses, hindering clinical translation of findings. Comparative studies have shown that differences in isolation methods can significantly impact EV yields and their microRNA contents, affecting the reproducibility of results [97]. Therefore, a thorough understanding of the advantages and disadvantages of each technique is crucial for selecting the most appropriate approach based on the aims of downstream applications.

Standardization efforts, such as those led by the International Society for Extracellular Vesicles, aim to establish guidelines for EV isolation and characterization to promote reproducibility and facilitate data comparison across studies [1,31]. These initiatives underscore the importance of optimizing isolation techniques and implementing quality control measures to ensure the clinical utility of EV-based diagnostics. Future research should focus on developing automated and scalable isolation methods that combine high efficiency with minimal sample processing time. Additionally, the integration of multi-omic approaches and artificial intelligence-driven data analysis could expedite the translation of EV and miRNA-based diagnostics and therapeutics to the clinic.

Several EV-based products are currently being assessed by clinical trials, indicating a growing interest in determining their real value for disease diagnosis and therapy with recent advancements in the field that will hopefully ensure timely and successful integration into clinical practice [169,278], a task that may be hindered by the limitations of current methods to track and characterize EVs [298]. An additional limitation for the timely translation to the clinic seems to be imposed by specific regulatory requirements differing from those of synthetic nanoparticles [299,300].

Employing artificial intelligence and machine learning in the design and engineering of EV-based therapeutics could accelerate clinical outcomes and facilitate translation of EV-based products [301]. The transition from research to commercialization often faces obstacles such as funding shortages and market competitiveness. Promoting academic entrepreneurship and collaboration between research and business sectors could help bridge this gap and encourage the development of EV-based therapeutics [302]. While the field of EVs has made significant advancements in recent decades, ongoing efforts in standardization, manufacturing, regulatory guidelines, and technological integration are essential for the translation of their full potential into clinical applications [31,169,278].

## 8. Conclusions

EVs and their encapsulated microRNAs have emerged as promising biomarkers for diagnostic applications, offering a minimally invasive approach for disease detection and monitoring. The ability of EVs to transport functional biomolecules across biological barriers underscores their potential utility in clinical settings. However, the success of EV-based diagnostics hinges on the development of reliable and reproducible isolation methodologies.

Despite the significant progress made in EV isolation techniques, the establishment of standardized methods granting consistent yields, high purity, and integrity of vesicles across different sample types and experimental conditions remains needed. The heterogeneity of EV populations and the complexity of biological fluids present additional challenges in achieving methodological uniformity. Standardizing EV isolation protocols is a critical goal for the field, as it will enable more accurate characterization of vesicle-associated microRNAs and enhance their diagnostic applicability. Future research efforts should focus on refining existing techniques and developing consensus guidelines to facilitate cross-study comparability and clinical translation.

On another side, the detection of miRNAs is a rapidly evolving field with significant implications for biomarker discovery, disease diagnostics, and therapeutic monitoring. While qRT-PCR, microarray analysis, NGS, ISH, NB, biosensors, ddPCR, and NanoString each offer unique strengths, none of them is considered better than the other. The selection of an optimal detection platform depends on factors such as sensitivity, specificity, cost, and throughput. Emerging technologies aim to address the current limitations by improving sensitivity, reducing cost, and making data interpretation easier with an expected significant impact in the advancement of this field.

## Figures and Tables

**Table 1:** Advantages and disadvantages summary of main EV isolation methods described in Section 3.

Isolation Method	Advantages	Disadvantages	References
UC	Large sample amounts can be obtained. Easy to use. Low processing costs, capacity to handle large sample volumes, simultaneous separation of multiple EV samples, and no additional reagents are needed. DGUC: EV subtype isolation capacity.	Requirement of specialized equipment. EV structure may be disrupted. EV loss, fusion, distortion, and co-isolation of contaminants. Requires large sample volumes. Time-consuming. Dependent on the rotor, the temperature, and viscosity of the sample. Exosome aggregation can be induced. DGUC: Additional purification procedures are required to remove gradient solution in downstream applications.	[70,71,72,73,77]
Precipitation	Does not require specialized equipment, is quick, simple, affordable, and the volume required is low. EVs are not damaged. High isolation yield.	Poor EV purity, and protein contamination is generally high (pretreatment with proteases might help).	[82,83]
Immunoaffinity	High purity of isolated EVs by a rather simple process.	EV structure can be impacted by the non-neutral pH and non-physiological elution buffers.	[93,98]
EV sorting	Enables high-throughput analysis and categorization of EV based on biomarker expression.	High costs for modifying equipment and time-consuming. Detection limits in particle size.	[102,103]
UF	Fast, simple, and quick. It does not require special equipment.	It can damage EVs from shear stress, particle aggregation might compromise EV yields and consistency.	[67,97,106]
SEC	EVs maintain their integrity, important for biological activity assessment assays. High yields and low contamination.	Optimized SEC columns according to sample volume and type are required.	[113,114]
Microfluidics	Very high purity and recovery rate. EVs maintain their biological function.	Time-consuming	[131]

**Table 2:** Examples of circulating miRNAs identified in the referenced studies as disease biomarkers; the detection methods used in the studies in which they were identified are included.

miR-15a and miR-16	Chronic Lymphocytic Leukemia	qRT-PCR	[208]
miR-21	Various Cancers (e.g., breast, lung, prostate)	qRT-PCR	[209]
miR-126	Lung Cancer	Microarray Analysis	[210]
miR-122	Hepatocellular Carcinoma	Northern Blot and qRT-PCR	[211]
miR-155	Diffuse Large B-Cell Lymphoma	qRT-PCR	[212]
miR-21, miR-126, miR-146a	COVID-19	qRT-PCR	[213]
miR-196b, miR-31, miR-891a, miR-34c, miR-653	Lung Adenocarcinoma	Transcriptome Analysis	[214]
miR-21, miR-155	Breast Cancer	Electrochemical Biosensors	[215]
miR-122, miR-192	Hepatocellular Carcinoma	NGS	[216]
miR-29a, miR-181b	Alzheimer’s Disease	Surface-Enhanced Raman Scattering (SERS) Biosensors	[217]

**Table 3:** Summary of strengths and weaknesses of each miRNA detection method described in Section 5.

Detection Method	Advantages	Disadvantages	Reference
qRT-PCR	High sensitivity and specificity. Quantitative and widely used.	Requires prior sequence knowledge. Limited detection of novel miRNAs.	[232]
Microarray Analysis	High-throughput detection of multiple miRNAs. Suitable for comparative expression profiling.	Lower sensitivity than qRT-PCR. Detects only known miRNAs.	[231]
NGS	Allows discovery of novel miRNAs. High sensitivity and dynamic range.	Expensive and requires complex bioinformatics. Long turnaround time.	[236]
ISH	Provides spatial distribution of miRNA expression. Single-cell resolution.	Less quantitative than qRT-PCR/NGS. Requires high-quality tissue samples.	[240,241]
NB	Confirms miRNA integrity and size.	Labor-intensive and requires large RNA amounts. Low sensitivity.	[245,248]
Biosensors	Rapid and real-time detection. Potential for portable diagnostics.	Requires careful design and optimization. May be affected by biological sample complexity.	[250,257,261,262]
ddPCR	Absolute quantification without a standard curve. High sensitivity, even for low-abundance miRNAs. Resistant to PCR inhibitors.	More expensive than qRT-PCR. Limited multiplexing capabilities.	[257,258]
NanoString	Direct and absolute quantification. High specificity due to sequence-specific probes. Multiplexing capability. Works well with low RNA input and degraded samples. High reproducibility and ease of use.	Lower sensitivity compared to qPCR for low-abundance miRNAs. Higher cost per sample compared to some qPCR-based methods. Requires specialized equipment (nCounter system).	[259,260]

## Data Availability

No new data were created or analyzed in this study. Data sharing is not applicable to this article.

## Abbreviations

The following abbreviations are used in this manuscript:

EV	Extracellular vesicle
miRNA	microRNA
MVBs	Multivesicular bodies
ESCRT	Endosomal Sorting Complexes Required for Transport
MSC	Mesenchymal Stem Cell
UC	Ultracentrifugation
dUC	Differential Ultracentrifugation
DGUC	Density Gradient Ultracentrifugation
PEG	Polyethylene Glycol
TEIR	Total Exosome Isolation Reagent
FSC	Forward Scattered
SSC	Side Scattered
sEV	Small extracellular vesicle
MWCO	Molecular Weight Cutoff
UF	Ultrafiltration
TFF	Tangential Flow Filtration
SEC	Size exclusion chromatography
RInSE	Rapid Inertial Solution Exchange
PEEK	Polyetheretherketone
TBS	Tris-Buffered Saline
IgG	Immunoglobulin G
pre-miRNA	miRNA precursor
DGCR8	DiGeorge Syndrome Critical Region 8
RISC	RNA-induced silencing complex
mRNA	messenger RNA
Ago2	Argonaute 2
RBP	RNA-binding protein
lncRNAs	long non-coding RNAs
UTR	Untranslated Region
ASO	Antisense Oligonucleotide
HCV	Hepatitis C Virus
qPCR	quantitative Polymerase Chain Reaction
qRT-PCR	Quantitative Real-Time PCR
RT	reverse transcription
cDNA	complementary DNA
NGS	Next-Generation Sequencing
ISH	In Situ Hybridization
smRNA	Single-molecule RNA
NB	Northern Blot
SERS	Surface-enhanced Raman scattering
EM	Electromagnetic Mechanism
CM	Chemical-enhancement Mechanism
PI-SPR	Phase Imaging Surface Plasmon Resonance
ddPCR	Digital Droplet PCR
MS	Mass Spectrometry
LC	Liquid Chromatography
DLS	Dynamic Light Scattering
NTA	Nanoparticle Tracking Analysis
AFM	Atomic Force Microscopy
TIRF-M	Total Internal Reflection Microscopy
SEM	Scanning Electron Microscopy
TEM	Transmission Electron Microscopy
cryo-TEM	Cryo-Transmission Electron Microscopy
TRPS	Tunable Resistive Pulse Sensing
IRIS	Interference Reflectance Imaging Sensor
SMLM	Single-Molecule Localization Microscopy
STED	Stimulated Emission Depletion
RS	Raman Spectroscopy
SERS	Surface-enhanced Raman scattering
TERS	Tip-Enhanced Raman Scattering
FT-IR	Fourier-Transform Infrared
FCS	Fluorescence Correlation Spectroscopy
micro-CT	Micro-Computed Tomography
ELISA	Enzyme-Linked Immunosorbent Assay
